# C-type natriuretic peptide moderates titin-based cardiomyocyte stiffness

**DOI:** 10.1172/jci.insight.139910

**Published:** 2020-11-19

**Authors:** Konstanze Michel, Melissa Herwig, Franziska Werner, Katarina Špiranec Spes, Marco Abeßer, Kai Schuh, Swati Dabral, Andreas Mügge, Hideo A. Baba, Boris V. Skryabin, Nazha Hamdani, Michaela Kuhn

**Affiliations:** 1 Institute of Physiology, University of Würzburg, Würzburg, Germany.; 2 Comprehensive Heart Failure Center, University Hospital Würzburg, Würzburg, Germany.; 3 Institute of Physiology and; 4 Department of Cardiology, St-Josef Hospital, Ruhr University Bochum, Bochum, Germany.; 5 Institute of Pathology, University Hospital Essen, University Duisburg-Essen, Essen, Germany.; 6 Medical Faculty, Core Facility TRAnsgenic animal and genetic engineering Models (TRAM), University of Münster, Münster, Germany.

**Keywords:** Cardiology, Cyclic nucleotides, Guanylate cyclase, Heart failure

## Abstract

Heart failure is often accompanied by titin-dependent myocardial stiffness. Phosphorylation of titin by cGMP-dependent protein kinase I (PKGI) increases cardiomyocyte distensibility. The upstream pathways stimulating PKGI-mediated titin phosphorylation are unclear. We studied whether C-type natriuretic peptide (CNP), via its guanylyl cyclase-B (GC-B) receptor and cGMP/PKGI signaling, modulates titin-based ventricular compliance. To dissect GC-B–mediated effects of *endogenous* CNP in cardiomyocytes, we generated mice with cardiomyocyte-restricted GC-B deletion (CM GC-B–KO mice). The impact on heart morphology and function, myocyte passive tension, and titin isoform expression and phosphorylation was studied at baseline and after increased afterload induced by transverse aortic constriction (TAC). Pressure overload increased left ventricular endothelial CNP expression, with an early peak after 3 days. Concomitantly, titin phosphorylation at Ser_4080_, the site phosphorylated by PKGI, was augmented. Notably, in CM GC-B–KO mice this titin response was abolished. TAC-induced hypertrophy and fibrosis were not different between genotypes. However, the KO mice presented mild systolic and diastolic dysfunction together with myocyte stiffness, which were not observed in control littermates. In vitro, recombinant PKGI rescued reduced titin-Ser_4080_ phosphorylation and reverted passive stiffness of GC-B–deficient cardiomyocytes. CNP-induced activation of GC-B/cGMP/PKGI signaling in cardiomyocytes provides a protecting regulatory circuit preventing titin-based myocyte stiffening during early phases of pressure overload.

## Abstract 2

C-type natriuretic peptide via GC-B/cGMP/PKGI signalling in cardiomyocytes attenuates titin-based cardiomyocyte stiffening during early phases of pressure-overload.

## Graphical abstract

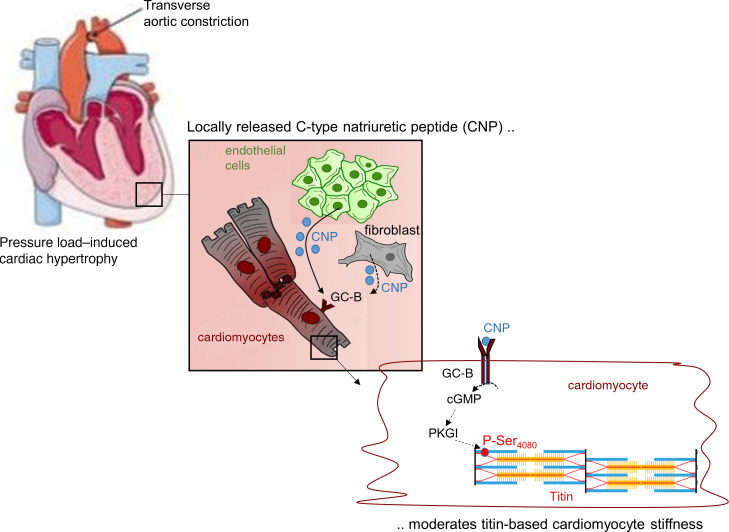

## Introduction

Increased myocardial passive stiffening and reduced chamber compliance accompany different heart diseases and are among the earliest characteristics of heart failure with preserved ejection fraction (HFpEF) (1, 2). Studies of samples from patients and experimental animals indicated that these changes are partly related to alterations of the giant sarcomeric protein titin (2–7). Titin acts as molecular spring spanning the sarcomere from the Z-disk to the M-line. It generates “passive” tension through its spring-like characteristics which determines the stiffness of the cardiomyocyte and thereby overall myocardial wall distensibility during diastolic filling (8, 9). The mechanical properties of titin depend on the proportion of 2 isoforms generated by alternate gene splicing: the shorter, stiffer N2B (molecular weight of ~3 MDa) and the longer, more compliant N2BA (~3.2 MDa). Furthermore, titin is modulated by posttranslational modifications, such as oxidation and phosphorylation of spring segments (6, 9). In particular, phosphorylation of specific residues within the unique sequence of the elastic spring region N2-Bus by cGMP-dependent protein kinase I (PKGI) decreases cardiomyocyte stiffness (9, 10). Consistently, PKGI activating interventions downward shifted the diastolic pressure-volume relationship during filling of intact hearts, indicating enhanced left ventricular (LV) distensibility (4, 5). Conversely, both PKGI activity and titin phosphorylation at the PKGI-specific site Ser_4099_ (corresponding to Ser_4080_ in murine titin) were markedly reduced in tissue from patients with heart failure, while passive myocyte stiffness was enhanced as compared with donor hearts (4, 5, 11). Together, these observations indicate that the second messenger cGMP, triggering PKGI activity, has an important role in the regulation of titin-dependent cardiomyocyte compliance. However, the upstream pathways enhancing cGMP/PKGI-dependent titin phosphorylation and thereby myocardial distensibility have not been clearly elucidated.

Cardiomyocytes’ cGMP levels and PKGI activity are modulated by 3 hormone-receptor systems. Nitric oxide (NO), mainly released from endothelial cells, stimulates the cytosolic “soluble” guanylyl cyclase. Atrial and B-type natriuretic peptide (ANP, BNP), released from cardiomyocytes, stimulate their shared transmembrane guanylyl cyclase–A (GC-A) receptor; and C-type natriuretic peptide (CNP), released from different types of cells in the heart, activates its specific transmembrane GC-B receptor (12, 13). In isolated rabbit cardiac muscle strips, pharmacological inhibition of NO signaling did not affect the decrease of passive tension in response to acute stretch. In contrast, this response was significantly reduced by blocking the actions of both NO and natriuretic peptides, an effect comparable to PKGI inhibition (14). Furthermore, in vivo, pharmacological activation of soluble guanylate cyclase did not influence ventricular compliance in normal and hypertrophied porcine hearts (15). Together these experimental studies indicate that the NO/cGMP signaling system is not solely involved in the PKGI-dependent regulation of titin-dependent chamber compliance. They suggest that natriuretic peptides may also regulate titin-based cardiomyocyte distensibility, but this has not been thoroughly investigated.

Despite sharing cGMP as a second messenger, the natriuretic peptides have distinct signaling and action patterns in cardiomyocytes. The GC-A receptors for ANP and BNP are confined to transverse tubules (T-tubules) and mediate small cGMP increases in local T-tubular microdomains (12, 16). GC-B receptors are more densely and uniformly distributed throughout the sarcolemma. Their activation by CNP generates greater cGMP signals spreading through the sarcoplasma and stimulating PKGI-mediated phosphorylation of regulatory proteins of the sarcoplasmic reticulum and the sarcomere, such as phospholamban and troponin I (16, 17).

The present studies aimed to characterize whether CNP, via GC-B/cGMP signaling, modulates titin isoform expression and phosphorylation and thereby LV compliance. We focused on CNP (instead of ANP/BNP) because most published studies on the cardiac roles of the CNP/cGMP pathway were performed with pharmacological application or transgenic overexpression of the peptide, thereby testing concentrations of CNP which were more than 1000-fold higher (100–1000 nM) than those occurring in peripheral or coronary blood (0.5–1 pM range) (18–24). To unravel the role of endogenously formed CNP in the regulation of titin, here we generated mice with a cardiomyocyte-restricted (CM-restricted) deletion of the GC-B receptor (CM GC-B–KO mice). The impact of ablated cardiomyocyte GC-B/cGMP signaling on LV morphology and function as well as on myocyte passive tension and titin isoform expression and phosphorylation was studied at baseline and after mild, short-time pressure overload. The results show that CNP enhances titin phosphorylation via the GC-B/cGMP/PKGI pathway and thereby moderates LV myocardial stiffness during early stages of enhanced afterload.

## Results

### Infusion of a low CNP dose enhances the phosphorylation of titin at the PKGI-specific site.

**Figure 1:**
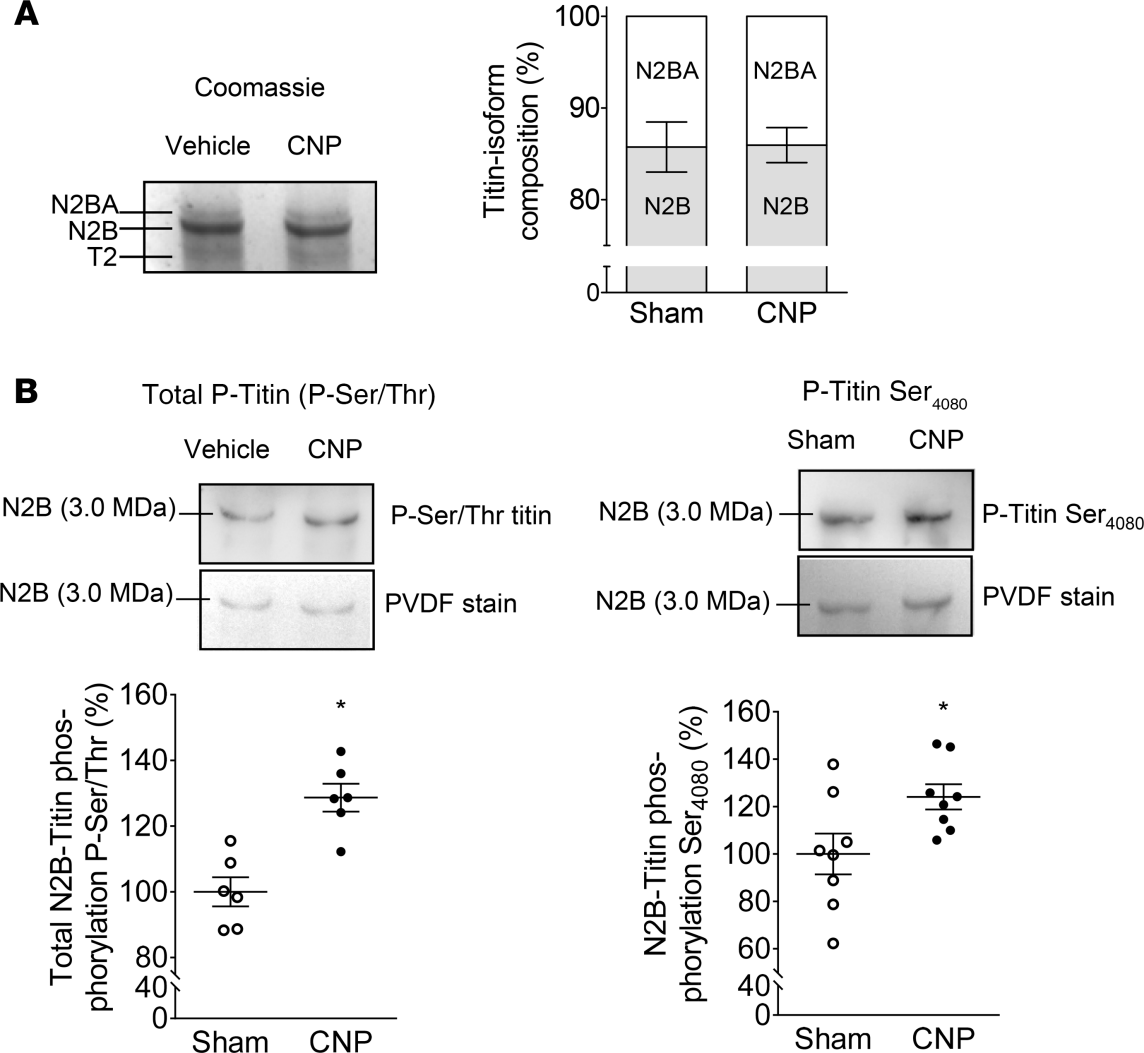
CNP infusion to mice did not change LV titin isoform composition but enhanced titin phosphorylation. (**A**) Titin gels (1.8% SDS-PAGE). (**B**) Western blot analyses using antibodies against total Ser/Thr-phosphorylated titin (left panel) and phospho-site–specific antibodies recognizing titin phosphorylated at Ser_4080_ (the PKGI-specific site; right panel). Data are shown as mean ± SEM. *n* = 6–8/group, *P < 0.05 versus sham (Student’s *t* test). T2, titin degradation; P, phospho.

As a proof of principle, to study CNP-dependent titin modifications firstly, *exogenous* synthetic CNP (0.05 μg/kg/min) was infused to WT C57BL6/N mice via osmotic minipumps during 2 weeks, raising circulating CNP plasma levels by about 1.5-fold (Supplemental Table 1; supplemental material available online with this article; https://doi.org/10.1172/jci.insight.139910DS1) (20). Published studies previously showed that at this dose CNP does not influence systemic arterial blood pressure (20). Using high-resolution gel electrophoresis, we evaluated LV titin N2BA/N2B isoform ratios in vehicle- versus CNP-treated mice but did not observe differences (Figure 1A). However, immunoblot studies revealed that CNP infusion significantly increased the phosphorylation of titin. Total serine/threonine titin phosphorylation as well as the phosphorylation of titin at Ser_4080_, the site specifically phosphorylated by PKGI (4, 5), were significantly greater in LV myocardium of CNP- as compared with vehicle-treated mice (Figure 1B). Supplemental Table 1 illustrates that such CNP infusions did not alter arterial blood pressure levels or LV systolic and diastolic functions. In particular, the LV end-diastolic pressure-volume relationship (EDPVR) was not altered by CNP under such baseline conditions of unaltered LV afterload.

### Cardiac CNP expression increases in response to increased pressure load.

**Figure 2:**
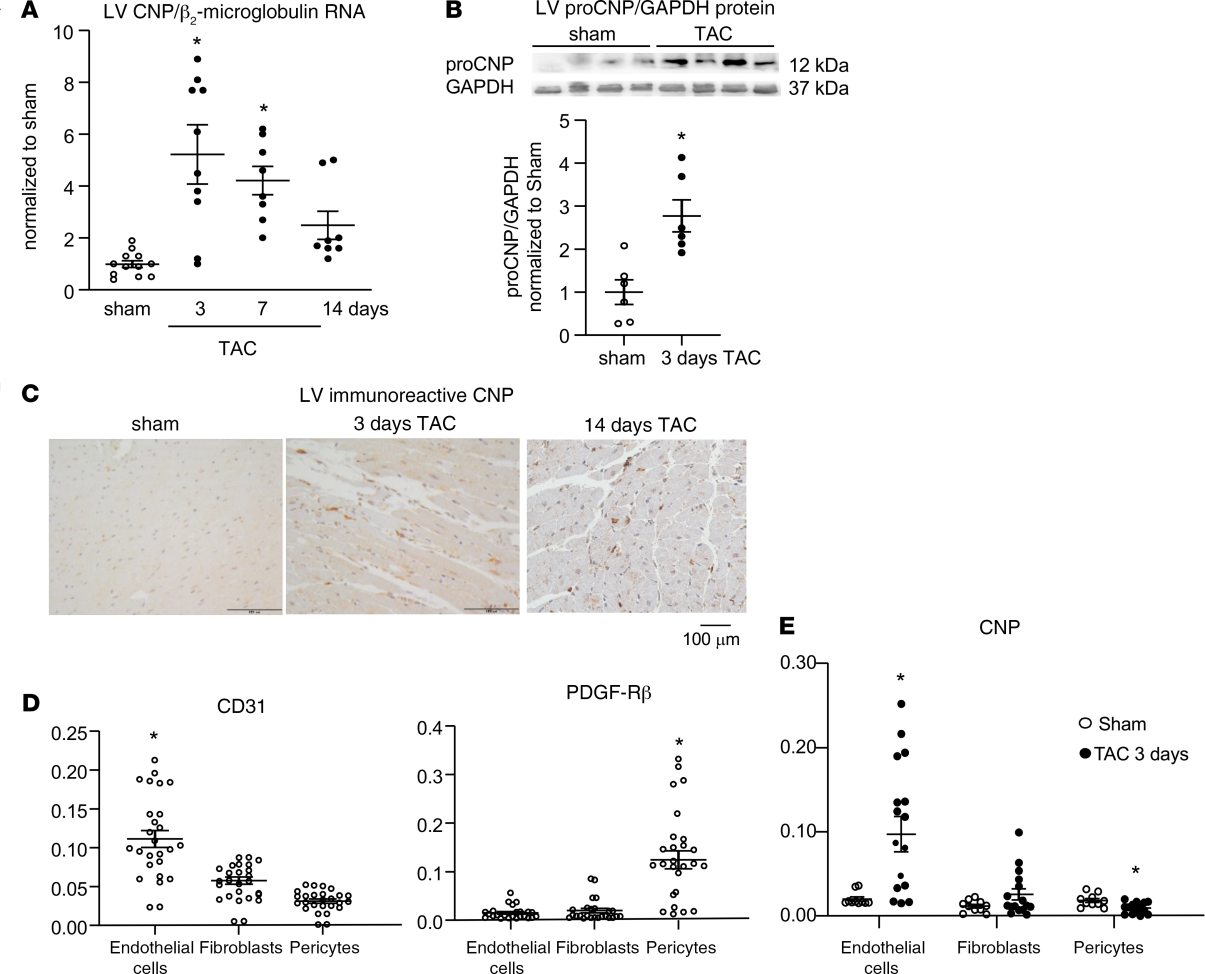
LV CNP expression increases in response to pressure overload provoked by TAC. (**A** and **B**) Real-time qRT-PCR and immunoblot: CNP mRNA levels were normalized to β_2_-microglobulin (**A**) and protein levels to GAPDH (**B**); ratios were calculated as x-fold versus sham. (**C**) Immunohistochemistry: representative pictures illustrate increased number of CNP-containing cells surrounding cardiomyocytes after TAC. (**D** and **E**) qRT-PCR: enrichment of cardiac CD31^+^ endothelial cells and PDGFR-β–positive pericytes with separation of PDGFR-α–positive fibroblasts (**D**) revealed TAC-induced CNP increases in endothelial cells (**E**). Target genes were normalized to β_2_-microglobulin. Data are shown as means ± SEM. *n* = 6–12 samples from 3–6 mice/group (in **A** and **B**), 26 samples from 13 mice (in **D**), and 10 sham (5 mice) versus 16 TAC (8 mice) (in **E**); *P < 0.05 vs. sham (**A**, **B**, and **E**) or other cell types (in **D**) (**A**: Kruskal-Wallis test; **B**: unpaired Student’s *t* test; **D**: 1-way ANOVA; and **E**: Mann-Whitney *U* test).

Our main goal was to study whether *endogenously* formed CNP regulates titin-dependent myocardial compliance under baseline or pressure overload conditions. To this aim, we firstly analyzed whether LV CNP expression in mice changes in response to mildly enhanced pressure load induced by surgical transverse aortic constriction (TAC) (17, 25). Quantitative real-time PCR (qRT-PCR) and immunoblot analyses showed significant increases of CNP expression after 3 days of TAC (mRNA by ~5-fold; protein ~3-fold; Figure 2, A and B). CNP levels reverted but stayed elevated after 7 and 14 days of TAC (Figure 2A). Concordantly, immunohistochemistry revealed an increase of CNP-containing cells around cardiomyocytes (Figure 2C).

To identify the types of cells involved in enhanced CNP production after TAC, we separated the nonmyocyte cell fraction from hearts of mice 3 days after sham or TAC surgeries into endothelial cells, pericytes, and fibroblasts by antibody- and magnetic-assisted cell sorting. CNP levels were studied by qRT-PCR. Figure 2D shows the enrichment of CD31^+^ endothelial cells and PDGFR-β–positive pericytes and separation of other cells, mostly fibroblasts. CNP mRNA was expressed in all 3 cell fractions, with lowest levels in fibroblasts (about 25% as compared with the levels of endothelia and pericytes) (Figure 2E). However, the peptide was significantly induced after TAC only in the endothelial cell enriched fraction (by 4.8 ± 1.05–fold vs. sham; *P* < 0.05). CNP mRNA expression was only mildly increased in fibroblasts (by 2.2 ± 0.34–fold vs. sham; *P* = 0.12) and even reduced in pericytes (by 52% ± 9%; *P* < 0.05) (Figure 2E).

### Generation of a genetic mouse model with cardiomyocyte-restricted GC-B deletion.

**Figure 3:**
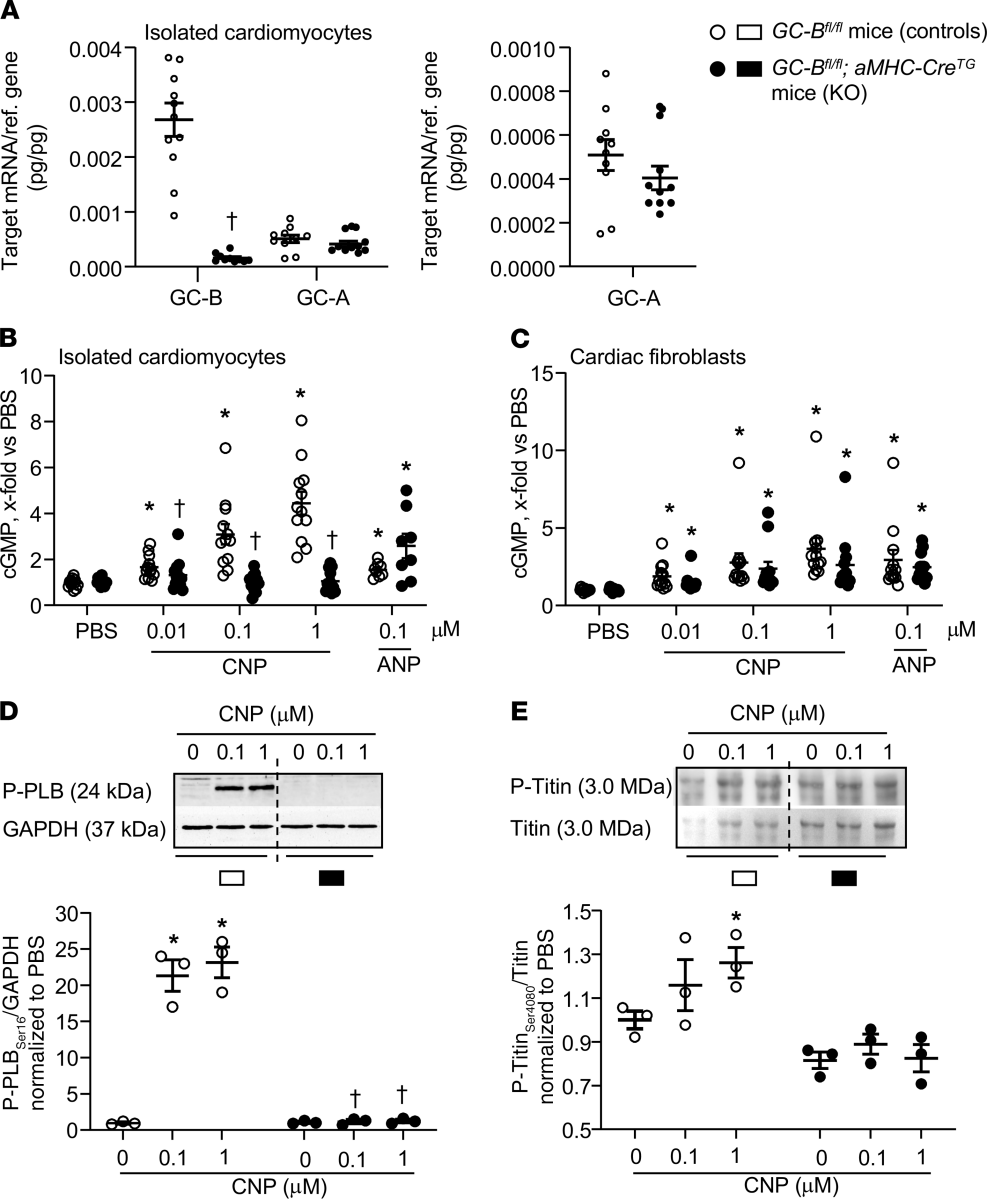
Abolished CNP/cGMP signaling in CMs from mice with CM-restricted deletion (KO) of the GC-B receptor (*GC-B^fl/fl^ αMHC-Cre^Tg^*). (**A**) qRT-PCR analyses. GC-B and GC-A mRNA expression levels in CMs isolated from *GC-B^fl/fl^* mice without (controls) or with the *αMHC-Cre^Tg^* (KO). Values are the ratio of target mRNA level relative to β_2_ microglobulin. *n* = 10 samples from 5 mice/genotype, ^†^*P* < 0.05 vs. *GC-B^fl/fl^* mice (Mann-Whitney *U* test). (**B** and **C**) CNP (10 nM to 1 μM) and ANP (100 nM) enhanced cGMP levels of control myocytes and fibroblasts. CNP effects were abolished in myocytes but preserved in fibroblasts from KO mice; ANP effects did not significantly differ between genotypes. *n* = 12 samples from 6 mice/genotype (2-way ANOVA). (**D** and **E**). Immunoblotting. In control myocytes, CNP increased the phosphorylation of phospholamban at Ser_16_ (**D**) and of titin at Ser_4080_ (**E**). In KO myocytes, these effects were abolished. (Top) Representative Western blots. (Bottom) Levels of phosphorylated phospholamban (P-phospholamban) pentamers were normalized to GAPDH and of P-titin to total titin. Ratios were calculated as x-fold respective vehicle samples. *n* = 3 samples/genotype. *P < 0.05 versus PBS (represented as 0), ^†^*P* < 0.05 versus control cells (2-way ANOVA). All data shown as means ± SEM.

To study whether *endogenous* CNP via cardiomyocyte GC-B/cGMP signaling regulates titin in vivo, we generated mice with a cardiomyocyte-targeted deletion of the GC-B receptor using *Cre/LoxP* technology. GC-B*^fl/fl^* mice (26) were mated to transgenic mice expressing Cre-recombinase under the control of the cardiac *αMHC* promoter (*αMHC-Cre^Tg^* mice; ref. 25). To assess the efficiency and selectivity of the GC-B deletion, we compared GC-B and GC-A expression and CNP- versus ANP-cGMP signaling in cardiomyocytes and fibroblasts prepared from GC-B*^fl/fl^* mice with and without the *αMHC-Cre* transgene. Figure 3A shows that GC-B mRNA expression in cardiomyocytes prepared from *GC-B^fl/fl^* *αMHC-Cre^Tg^* mice was almost fully abolished. As compared with GC-B, GC-A levels were much lower and not different between *GC-B^fl/fl^* and *GC-B^fl/fl^* *αMHC-Cre^Tg^* cardiomyocytes (Figure 3A, and amplification in right panel). CNP increased cGMP levels in myocytes from the *GC-B^fl/fl^* mice but not in myocytes from *GC-B^fl/fl^* *αMHC-Cre* mice (Figure 3B). In contrast, the effects of CNP on cGMP contents of cardiac fibroblasts as well as the effects of ANP on cardiac myocytes’ and fibroblasts’ cGMP levels were not different between the 2 genotypes (Figure 3, B and C).

Accordingly, in cardiomyocytes from the *GC-B^fl/fl^* mice, CNP led to PKGI-dependent phosphorylation of phospholamban at Ser_16_ and of titin at Ser_4080_; such effects were abolished in myocytes isolated from their *GC-B^fl/fl^* *αMHC-Cre* littermates (see immunoblots in Figure 3, D and E). Together these results demonstrate the deletion of GC-B in cardiomyocytes from *GC-B^fl/fl^* *αMHC-Cre* mice (hereafter referred to as CM GC-B–KO mice).

In contrast to mice with conventional, systemic GC-B deletion (27), such CM GC-B–KO mice have normal Mendelian inheritance, life span, and skeletal growth. Under resting conditions, arterial blood pressure, heart weights (HWs), as well as the ratio to BW (HW/BW) or tibia length (HW/tibia length) were not different between CM GC-B–KO and control (*GC-B^fl/fl^*) littermates. Invasive hemodynamic measurements showed normal LV contraction and relaxation (Supplemental Table 2 depicts the data obtained in 4-month-old males).

### CM GC-B–KO mice have unaltered heart remodeling after mild TAC.

To study whether *endogenous* CNP, via GC-B signaling, modulates the early responses of cardiomyocytes to mild pressure overload, we compared heart morphology and function of CM GC-B–KO and their control littermates after 3 and 14 days of TAC. We focused on these early stages based on the above-described CNP expression pattern. Since the cardiac parameters of respective sham-operated mice did not differ between these 2 time points, their results were combined.

**Figure 4:**
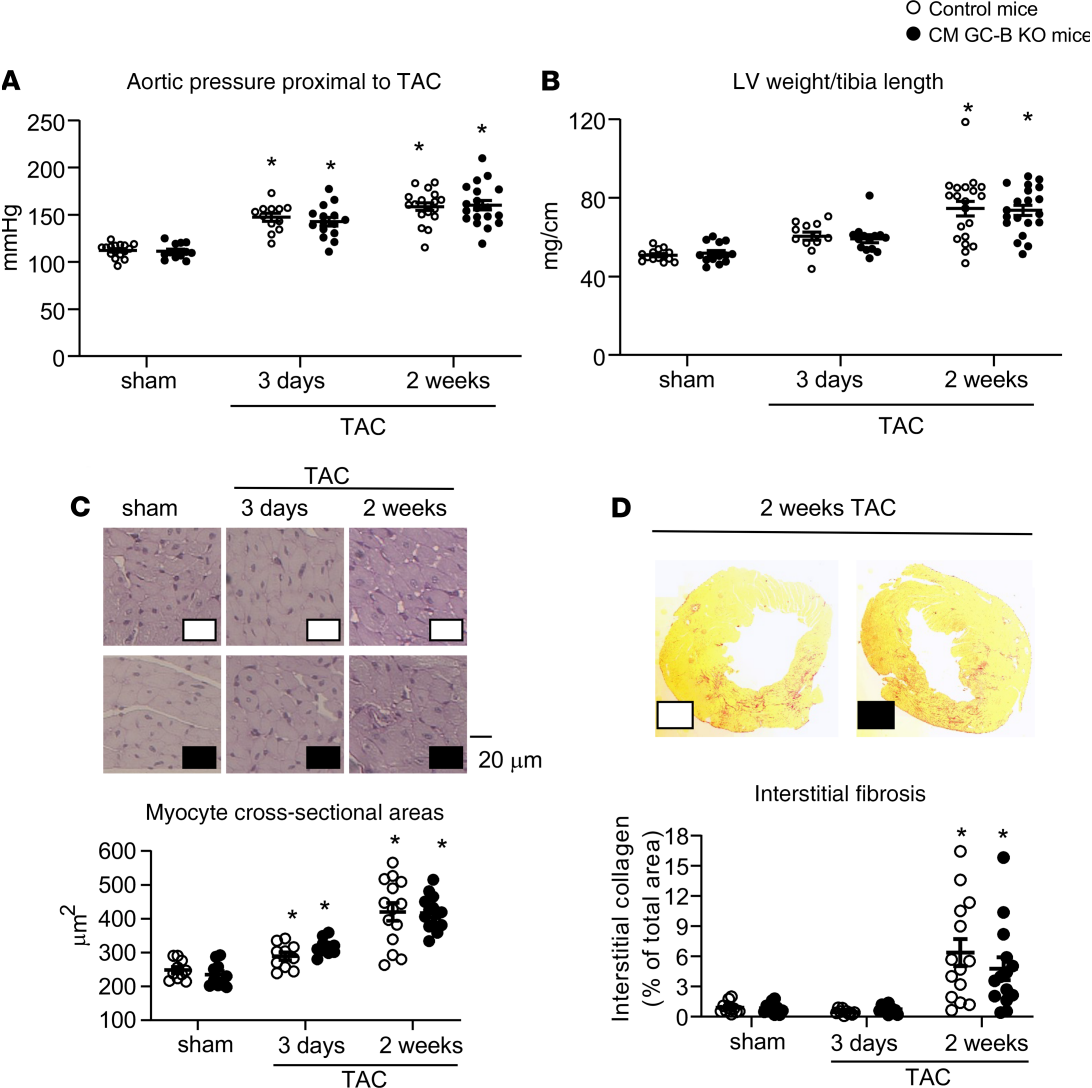
Pressure overload–induced LV remodeling was not different in control and CM GC-B–KO littermates. (**A**) Systolic blood pressure in the ascending aorta, proximal to the TAC, measured by retrograde catheterization in anesthetized mice. (**B**) LVW/tibia length ratios. (**C**) LV CM cross-sectional areas (90–120 cells per heart were measured). (**D**) LV interstitial collagen fractions (3 sections per mouse). Data are shown as mean ± SEM. *n* = 11–19/group, *P < 0.05 versus sham (**A**, **C**, and **D**, tested by 2-way ANOVA with Bonferroni’s multiple comparison *t* test; **B** by Kruskal-Wallis analysis).

Invasive pressure measurements in the aorta proximal to the stenosis confirmed that TAC provoked rapid (by 31 ± 4 mmHg, at 3 days) and incremental (48 ± 5 mmHg, at 14 days) increases in LV pressure load, without genotype-dependent differences (Figure 4A). As compared with sham mice, the LV weight (LVW) to tibia length ratios and cardiomyocyte areas were barely increased at 3 days but markedly augmented at 2 weeks of TAC, indicating progressive LV hypertrophy. Such hypertrophic responses were similar in control and CM GC-B–KO littermates (Figure 4, B and C). At 2 weeks of TAC, LV hypertrophy was accompanied by significant interstitial fibrosis, again without differences between the 2 genotypes (Figure 4D).

### CM GC-B–KO mice show LV dysfunction after mild pressure overload.

**Figure 5:**
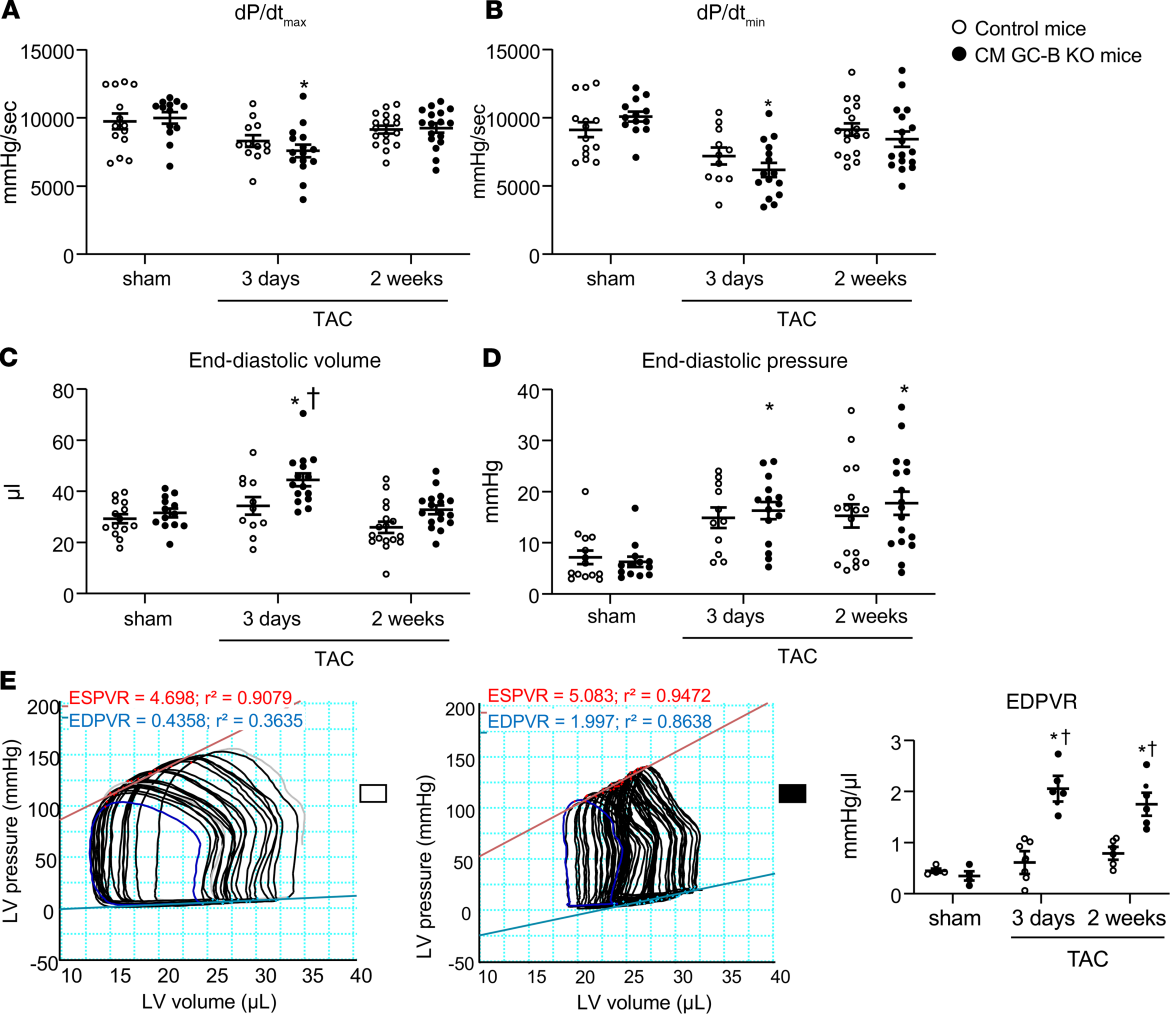
CM GC-B–KO mice with TAC during 3 or 14 days developed mild systolic and diastolic dysfunction and LV stiffening. (**A** and **B**) Invasive catheterizations showed that the maximal rates of LV contraction (**A**) and relaxation (**B**) were almost fully preserved in control mice subjected to 3 and 14 days of TAC but mildly impaired in CM GC-B–KO mice (in comparison with respective sham mice). (**C**–**E**) End-diastolic volumes (**C**) and pressures (**D**) as well as EDPVRs (**E**) were unaltered in control but increased in CM GC-B–KO mice with TAC. Data are shown as mean ± SEM. *n* = 11–19/group (**A**–**D**) or 4–7/group (**E**); *P < 0.05 versus sham, ^†^*P* < 0.05 versus control mice. (**A** and **D**: Kruskal-Wallis analysis; **B**, **C**, and **E**: 2-way ANOVA with Bonferroni’s multiple comparisons).

LV function was studied by invasive pressure-volume loop measurements in anesthetized mice (closed chest) (17, 26). In control mice with 3 or 14 days of TAC LV contraction and relaxation were only mildly impaired (as compared with sham mice). This was indicated by the small but not significant decreases of the LV maximal contraction (*dP/dt_max_*) and relaxation rates (*dP/dt_min_*) (Figure 5, A and B), ejection fractions, stroke volumes, and cardiac output (Supplemental Table 3). In the CM GC-B–KO mice the changes of these functional parameters after TAC were more pronounced. Hence, all parameters were significantly different from respective sham-operated mice of the same genotype, especially at 3 days of TAC (Figure 5, A and B; and Supplemental Table 3). Together these data show that pressure overload provoked mild LV dysfunction in the CM GC-B–KO mice, especially at very early time points (after 3 days of TAC). Nevertheless, the differences between genotypes were not statistically significant.

However, LV pressure-volume measurements during diastolic filling revealed striking differences between the 2 genotypes. Whereas in control mice after 3 and 14 days of TAC the end-diastolic volumes were not significantly altered (as compared with sham), in the KO mice with 3 days TAC they were significantly enlarged (Figure 5C). Moreover, only the KO mice showed enhanced end-diastolic pressures, indicating compromised ventricular filling (Figure 5D). To determine the EDPVR more precisely, transient inferior vena cava occlusions were performed during simultaneous measurements of LV pressure/volume loops. Whereas in the control mice with 3 or 14 days of TAC the EDPVRs were not different from sham, in their CM GC-B–KO littermates the EDPVRs were markedly increased at both study time points, indicating LV stiffness (Figure 5E illustrates these genotype- and condition-dependent differences).

### Pressure overload–provoked diastolic dysfunction of CM GC-B–KO mice is accompanied by diminished PKGI-dependent phosphorylation of titin.

**Figure 6:**
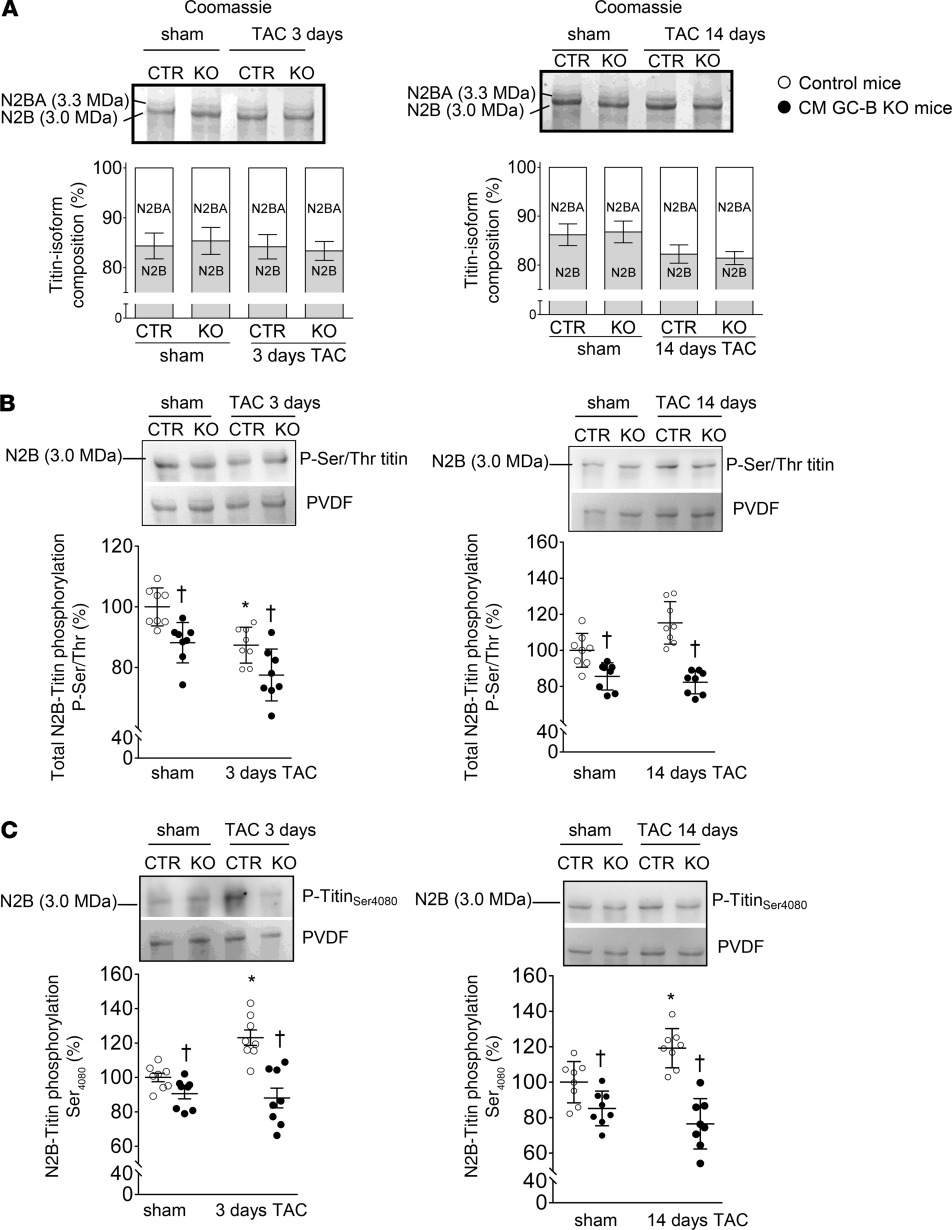
TAC increased the LV levels of Ser_4080_-phosphorylated titin in control but not in CM GC-B–KO mice. (**A**) Titin gels (1.8% SDS-PAGE) revealed that titin isoform composition was not altered by genotype or condition. (**B**) Western blot analyses using antibodies against total Ser/Thr-phosphorylated titin. (**C**) Phospho-site–specific antibodies recognizing titin phosphorylated at Ser_4080_ (the PKGI-specific site). The levels of phosphorylated titin were normalized to total titin (PVDF) and calculated as percentage (%) from sham control mice. Data are shown as mean ± SEM. *n* = 8 mice/group tested in duplicates. *P < 0.05 versus sham-operated mice; ^†^*P* < 0.05 versus control (*GC-B^fl/fl^*) mice (2-way ANOVA).

Using high-resolution gel electrophoresis, we evaluated the LV titin N2BA/N2B isoform ratios in sham versus TAC mice but did not observe TAC- or genotype-dependent changes (Figure 6A). In both control and KO mice, the proportion of the longer, more compliant N2BA isoform slightly increased at 14 days of TAC, but this adaptive response did not reach statistical significance.

We performed immunoblot studies using antibodies that recognize either total serine/threonine titin phosphorylation, Ser_4080_-phosphorylated titin (the site phosphorylated by PKGI), or Ser_3991_-phosphorylated titin (corresponding to Ser_4010_ in human titin, a site phosphorylated both by cAMP-dependent (PKA) and extracellular signal–regulated (ERK2) kinases (28–30). In control mice the LV levels of total Ser/Thr-phosphorylated titin were slightly diminished at 3 days and increased at 14 days of TAC (Figure 6B). In contrast, the selective phosphorylation of titin at Ser_4080_ was significantly enhanced at both time points as compared with sham mice (Figure 6C). The phosphorylation of titin at Ser_3991_ was also mildly enhanced at 3 and 14 days of TAC, although without reaching statistical significance (Supplemental Figure 1A). Notably, in their CM GC-B–KO littermates the LV levels of total Ser/Thr-phosphorylated titin and of Ser_4080_-phosphorylated titin were significantly reduced to some extent under sham and even more under TAC conditions (Figure 6, B and C). In other words, the TAC-induced increases of P-titin-Ser_4080_ observed in controls were fully absent in such KO littermates (Figure 6C). In contrast, the LV levels of Ser_3991_-phosphorylated titin were significantly enhanced in the KO mice to some extent under sham conditions and even more after TAC (Supplemental Figure 1A).

It was shown that *exogenous* CNP, via GC-B/cGMP signaling, counterregulates ERK activation in cultured cardiomyocytes (31). Therefore we followed the hypothesis that, conversely, increased ERK activity contributed to enhanced LV Ser_3991_-titin phosphorylation in CM GC-B–KO mice. Indeed, the LV levels of both total and Thr_185_/Tyr_187_-phosphorylated ERK2 were significantly increased in the KO mice to some extent under sham conditions and even more after TAC (as compared with control mice) (Supplemental Figure 1B). The ratios of phosphorylated/total ERK did not differ between the 2 genotypes.

### CM GC-B–KO mice showed diminished left ventricular levels of phosphorylated phospholamban and troponin I.

It was reported that high concentrations of *exogenous* CNP stimulate the phosphorylation of phospholamban at Ser_16_ and of troponin I at Ser_23/24_ and thereby myocyte lusitropy (17, 22). To follow up the role of the *endogenous* hormone, we compared the LV expression and phosphorylation of these regulatory proteins in control and CM GC-B KO mice. The total levels of phospholamban (Supplemental Figure 2A, left) and troponin I (Supplemental Figure 2B, left) were not different between genotypes and conditions. However, the LV levels of Ser_16_-phosphorylated phospholamban (Supplemental Figure 2A, right) and of Ser_23/24_-phosphorylated troponin I (Supplemental Figure 2B, right) were mildly but significantly reduced in the KO mice to some extent under sham conditions and even more after TAC (as compared with respective CTR mice).

### Skinned LV fibers prepared from CM GC-B–KO mice with TAC have increased passive tension.

**Figure 7:**
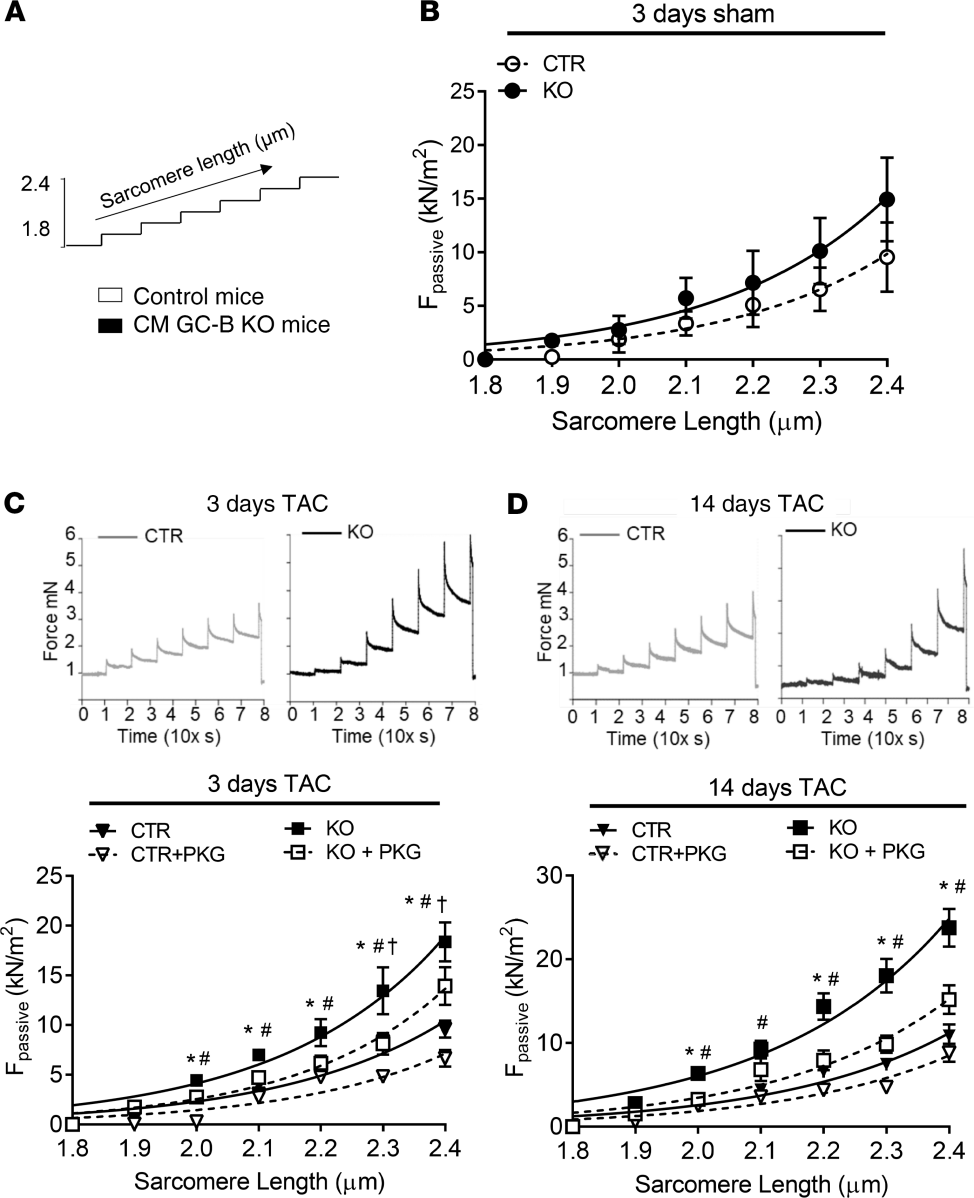
Isolated LV skinned CMs from CM GC-B–KO mice with TAC showed enhanced stiffness, which was reversed by addition of recombinant PKGI. (**A**) Stretch protocol used in these experiments (sarcomere lengths 1.8–2.4 μm). (**B**–**D**) Single skinned CMs were prepared from sham control (CTR) and CM GC-B–KO (KO) mice (**B**) and from both genotypes after 3 days (**C**) or 14 days (**D**) of TAC for recordings of the force response to stepwise cell stretching in relaxing buffer. Top panels in **C** and **D**: examples of original recordings. (Bottom) In addition passive force (F_passive_) in relation to sarcomere length was recorded for control and GC-B–KO CMs from mice with TAC without or with pretreatment with recombinant PKGI. Data are shown as means ± SEM. *n* = 16–24 CMs from 4–5 hearts per group. *P < 0.05 KO versus CTR (1-tailed Student’s *t* test); ^#^*P* < 0.05 KO without and with PKGI (2-tailed Student’s *t* test); ^†^*P* < 0.05 CTR without and with PKGI (2-tailed Student’s *t* test).

To study the functional consequences of diminished PKGI-mediated titin phosphorylation in LV myocardium from CM GC-B–KO mice, we compared the passive force–sarcomere length relation of single skinned myocytes prepared from control and KO mice (sham versus TAC) using a stretch protocol (Figure 7A). The mean values were fitted with a second-order polynomial curve (Figure 7, B–D). Figure 7B depicts that the passive force–sarcomere length relations were similar for skinned myocytes from sham control and KO mice. Also, skinned cardiomyocytes prepared from control mice with 3 or 14 days of TAC had unaltered passive force–sarcomere length relations (Figure 7, C and D). In contrast, the cardiomyocytes prepared from KO mice with TAC developed markedly increased passive force for sarcomere lengths in the range of 2.0 to 2.4 μm as compared with cells from control mice with TAC, indicating diminished elasticity (Figure 7, C and D).

**Figure 8:**
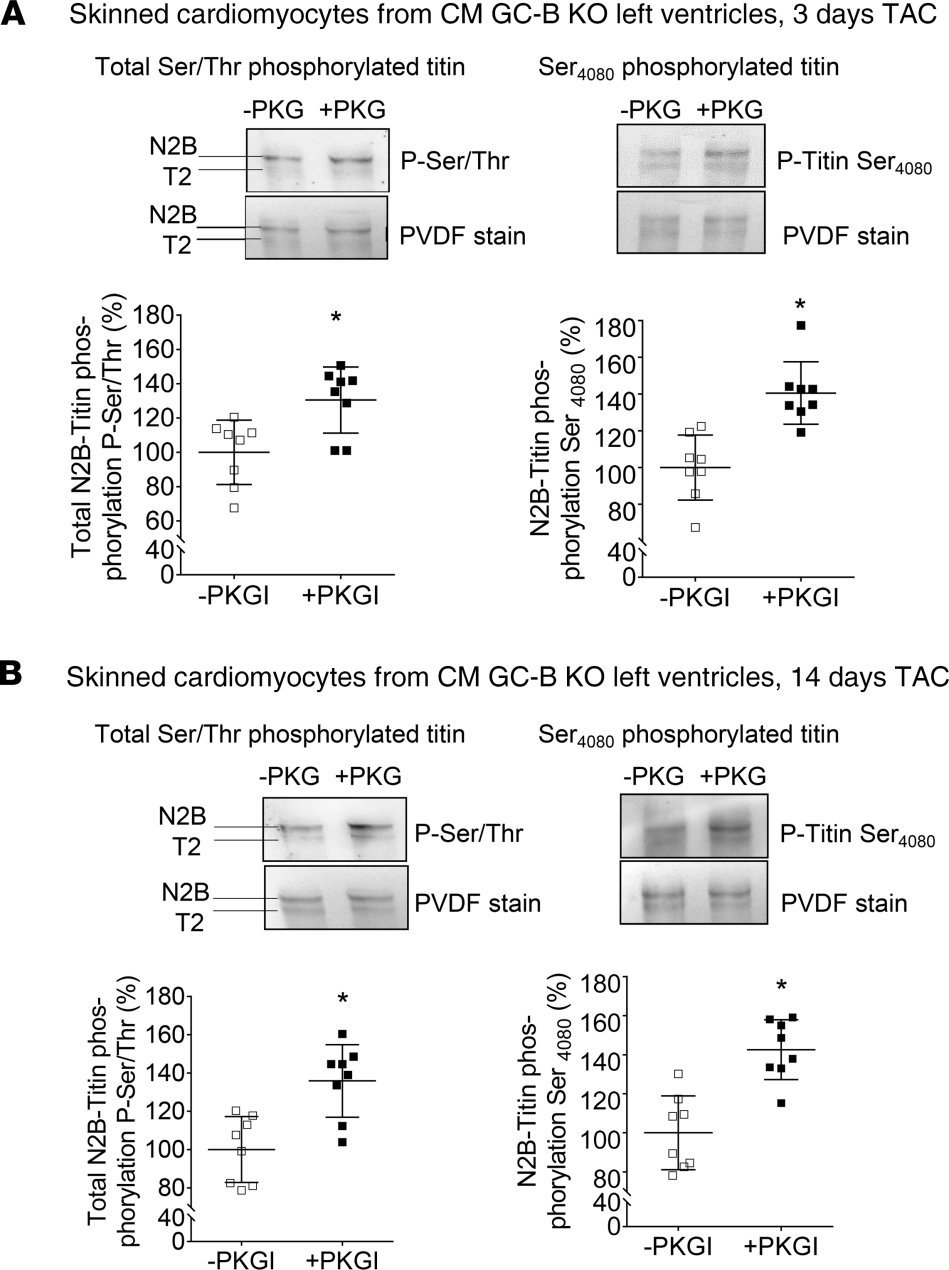
Addition of recombinant PKGI enhanced total Ser/Thr- and Ser_4080_-titin phosphorylation in skinned CMs prepared from CM GC-B–KO mice after TAC. LV CMs were prepared from CM GC-B–KO mice after 3 days (**A**) or 14 days (**B**) of TAC. Western blot analyses were performed using an antibody against total Ser/Thr-phosphorylated titin (left panel) and a phospho-site–specific antibody recognizing titin phosphorylated at Ser_4080_ (the PKGI-specific site) (right panel). The levels of phosphorylated titin were normalized to total titin (PVDF) and calculated as percentage (%) from the mean value of the untreated (-PKGI) sample group. Data are shown as means ± SEM. *n* = 8/group, *P < 0.05 versus untreated samples (-PKGI) (2-tailed Student’s *t* test).

To test whether the increased passive tension of skinned myocytes prepared from CM GC-B KO mice with TAC was related to the reduced PKGI-mediated titin phosphorylation, these experiments were repeated after treatment with exogenous, recombinant PKGI (dashed lines in Figure 7, C and D). In skinned myocytes from control mice with TAC, exogenous PKGI barely affected passive stiffness, and the effect was seen only at very high sarcomere lengths of 2.3 and 2.4 μm (Figure 7, C and D). In contrast, in myocytes from CM GC-B–KO mice with TAC, the incubation with PKGI reduced stiffness significantly (Figure 7, C and D). Concomitantly, PKGI rescued the attenuated levels of total Ser/Thr- and Ser_4080_- phosphorylated titin in skinned myocytes prepared from CM GC-B–KO mice with 3 or 14 days of TAC (Figure 8, A and B). Together these results reveal that the cardiomyocytes of CM GC-B–KO mice react to mild pressure overload with enhanced passive stiffness. They suggest that PKGI reverses this phenotype via a direct effect on sarcomeres, presumably on titin.

## Discussion

Molecular alterations of titin and enhanced titin-based cardiomyocyte stiffness contribute to diastolic dysfunction in heart failure (1–7). Studies of LV samples from patients with dilated cardiomyopathy or hypertrophic cardiomyopathy or with HFpEF associated with type 2 diabetes indicated that diminished phosphorylation of titin by PKGI contributes to these changes (3, 10, 11, 15, 32). However, the upstream pathways normally stimulating PKGI-dependent titin phosphorylation in vivo remain unclear. Our pharmacological and genetic studies in mice demonstrate that CNP, via GC-B/cGMP signaling in cardiomyocytes, augments PKGI-mediated phosphorylation of the titin springs at the specific site Ser_4080_ (corresponding to Ser_4099_ in human titin; ref. 10). This effect does not modulate LV compliance under physiological conditions of normal cardiac pressure load but prevents increased myocyte stiffening and diastolic dysfunction during early stages of mild pressure overload–induced cardiac hypertrophy.

### Cardiac pressure overload increases endothelial CNP expression.

Increased afterload (here induced by TAC) augmented cardiac CNP expression, with an early peak at 3 days after TAC. In accordance with published studies (12, 13, 33), we observed that fibroblasts, endothelial cells, and additionally pericytes might be local sources of the peptide under these conditions. Our studies of enriched types of cells support the notion that the increased cardiac expression of CNP after 3 days of TAC is mainly contributed by endothelial cells. Inflammation, cytokines, and hypoxia might induce endothelial CNP expression under such conditions (12). Supporting the notion that CNP is an “acute-phase” reactant, in rats with experimental myocardial infarction, CNP expression increased on day 3 after infarction by about 4-fold in the infarcted LV and gradually decreased from day 7 to day 18 (34). Conversely, cardiac CNP expression levels were diminished in samples from patients with end-stage heart failure (35). Together with these published results our observations suggest that locally formed, paracrine-acting coronary endothelial CNP might protect cardiomyocytes’ functions in situations of acute “stress,” such as pressure load or ischemia.

### CNP modulates titin phosphorylation and titin-based cardiomyocyte stiffness.

Concomitantly to the increased CNP expression, PKGI-dependent phosphorylation of titin at Ser_4080_ was markedly increased in LV myocardium early after TAC. Notably in mice with ablated CNP/GC-B signaling in cardiomyocytes (CM GC-B–KO mice), this titin response was abolished. Accordingly, such KO mice reacted to mild TAC (increasing afterload by ~30-50 mmHg) with rapid LV myocyte stiffness and diastolic dysfunction, which was not observed in their control littermates. In vitro, recombinant PKGI rescued titin-Ser_4080_ phosphorylation and stiffness of GC-B–KO myocytes. Together these results indicate that activation of GC-B/cGMP/PKGI signaling in cardiomyocytes provides a protecting local regulatory circuit, which prevents titin-based myocyte stiffening during early phases of pressure overload. Titin springiness dominates the passive tension development in the mammalian heart in the sarcomere range from 1.9 to 2.2 μm, whereas collagen stiffness dominates at higher sarcomere lengths (6–8). This fact can explain our observation that CM GC-B–KO mice subjected to TAC had stiffer hearts than control littermates despite similar interstitial fibrosis.

Considering the huge size of the I-band titin spring, (de)phosphorylation of only 1 site may have a negligible effect on overall titin-based tension. It is more likely that several sites within the N2-Bus and/or other titin spring segments must be modified at one time by a specific signaling pathway to result in a mechanical effect. In line with this assumption we observed that CNP infusions in vivo enhanced not only the phosphorylation of titin at Ser_4080_ but also total serine/threonine titin phosphorylation, implying that several sites were modified. Conversely, both Ser_4080_ and overall serine/threonine titin phosphorylation were diminished in CM GC-B–KO mice. Notably, besides activating PKGI, CNP-induced cGMP signaling inhibits the phosphodiesterase 3A–mediated degradation of cAMP. This positive cGMP-to-cAMP crosstalk can augment the activity of cAMP-dependent protein kinase (PKA) in cardiomyocytes (22). PKA phosphorylates Ser_3991_ within the N2-Bus region (corresponding to Ser_4010_ in human titin), and the phosphorylation of this site has also been associated with a decrease in titin-based tension (9). Therefore we considered that reduced Ser_3991_-titin phosphorylation contributed to the alterations of CM GC-B–KO mice. Unexpectedly, immunoblots with a phosphospecific antibody revealed increased (not decreased) LV levels of Ser_3991_-phosphorylated titin in KO mice both under sham and under TAC conditions. The site Ser_3991_ is phosphorylated not only by PKA but also by ERK1/2 (28–30). On the other hand, it was shown in cultured neonatal rat cardiomyocytes that *exogenous* CNP attenuates basal and endothelin-1–induced hypertrophy-related gene expression and ERK phosphorylation (31). In line with these observations, LV expression and phosphorylation of ERK were mildly enhanced in CM GC-B–KO mice under sham and TAC conditions, which may have contributed to enhanced Ser_3991_-titin phosphorylation. It is presently unclear why this did not prevent stiffening of GC-B–KO myocytes after TAC and also why increased ERK did not result in enhanced hypertrophic responses.

Together our data suggest that CNP enhances PKGI- and counterregulates ERK-mediated phosphorylation of the titin springs. Additional unidentified PKGI-dependent phospho-sites may exist in cardiac titin and participate in the effect of CNP on cardiomyocyte stiffness (and in the stiffening of CM GC-B–KO hearts). This possibility is supported by our observation that recombinant PKGI enhanced not only Ser_4080_ but also overall Ser/Thr-titin phosphorylation in isolated skinned LV fibers (Figure 8).

### CNP moderation of cardiac stiffness is apparent only under pathological conditions.

It is noticeable that the CNP-induced changes in titin phosphorylation were only associated with diminished myocardial stiffness under conditions of pressure overload: in WT mice CNP infusions enhanced titin phosphorylation but did not change EDPVRs of normal hearts (Supplemental Table 1); conversely, in CM GC-B–KO mice, the levels of P-titin were diminished, but LV EDPVRs (in vivo) and myocyte passive force-to-sarcomere lengths (ex vivo) were not altered under sham conditions (Figure 5E and Figure 7B). This suggests that the CNP-induced phosphorylation(s) of titin mainly prevents increases in titin stiffness when titin is additionally modified by disease. For instance, published experimental studies suggested a link between the activation of renin-angiotensin-aldosterone system and increased titin-based stiffness through PKC-α–mediated hyperphosphorylation of titin’s PEVK element (made up of protein motifs rich in proline, glutamate, valine, and lysine). Such posttranslational modifications contribute to enhance cardiomyocyte passive tension and thereby to LV diastolic dysfunction (36, 37). In future studies we will follow the hypothesis that CNP/cGMP/PKGI signaling may counterregulate angiotensin/PKC-α–induced titin stiffness.

A majority of published experimental models of cardiac disease (myocarditis, diabetes, ischemia) presented with reduced total titin phosphorylation and increased myocardial stiffness compared with healthy controls, possibly driven by hypophosphorylation of residues in the N2-Bus segment (reviewed in ref. 9). In the present study the control mice exposed to mild LV pressure overload showed increased CNP expression, enhanced phosphorylation of titin at Ser_4080_, and unaltered LV compliance. These apparently discrepant results to previously published studies might be related to the stage of heart disease, the extent and persistence of experimental injury, and strain and species differences. Specifically, the here-reported alterations of CM GC-B–KO mice indicate that cardiomyocyte CNP/GC-B signaling has a critical role in the modulation of titin and prevention of diastolic dysfunction in situations of acutely increased cardiac afterload.

### Locally formed CNP regulates phospholamban and troponin I phosphorylations.

Previous studies in cultured cardiomyocytes and isolated perfused hearts indicated that CNP, via GC-B signaling and activation of PKGI or PKA, stimulates the phosphorylation of other regulatory proteins, such as phospholamban and troponin I, thereby improving diastolic function (17, 22). However, these studies were performed with very high pharmacological concentrations of CNP. Supporting a GC-B–mediated modulatory role of the *endogenous* hormone, we observed diminished LV levels of phosphorylated phospholamban and troponin I in CM GC-B–KO mice to some extent under sham and even more under TAC conditions. These molecular changes may have contributed to the mild pressure load–evoked LV dysfunction of such KO mice. The exact mechanisms and implications are outside of the scope of our here-presented titin-focused studies.

### Therapeutical relevance.

Neuroendocrine imbalance, with increased activity of the renin-angiotensin-aldosterone and sympathetic systems and diminished activity of the natriuretic peptides, has a major role in the transition of cardiac hypertrophy to heart failure (12, 38). Blockade of angiotensin II signaling together with inhibition of neprilysin-mediated degradation of the natriuretic peptides by the drug LCZ696 (Entresto^R^) has improved the morbidity and prognosis of heart failure patients (39). Notably, neprilysin degrades CNP more than ANP/BNP (12, 40), but the contribution of augmented *endogenous* CNP signaling to the clinical benefits of neprilysin inhibitors is not known. Despite recent setbacks in ongoing studies of LCZ696 in heart failure patients with overly stiff hearts (such as HFpEF; ref. 41) the results of the here-presented focused experimental studies suggest that correction or augmentation of CNP/titin signaling may moderate pathological myocardial wall stiffness and improve diastolic function at earlier or less severe disease stages. The concept that augmentation of CNP bioactivity may have a clinical benefit is supported by the recent experimental findings that CNP exerts not only GC-B/cGMP-mediated but also cGMP-independent protective actions on cardiomyocytes (13). These actions involve a second CNP receptor, the G_i_-coupled natriuretic peptide receptor C (NPR-C) (13). In addition CNP, via GC-B and possibly also NPR-C signaling, acts on other types of cells in the heart, such as fibroblasts, immune cells, and vascular cells (12, 13, 18–21, 34). Thereby CNP may not only moderate cardiomyocyte stiffness and dysfunction (present studies) but also improve coronary perfusion and attenuate the hypertrophic, fibrotic, and inflammatory responses of the heart to ischemia and hypertension (12, 13, 42).

### Study limitations.

Because the effects of cardiomyocyte GC-B deletion were evaluated after 3 days and 2 weeks of pressure overload in the present study, for future clinical application, further studies are necessary to examine if the effects of CNP on myocardial compliance persist for longer term follow-up periods.

## Methods

### CNP infusions.

Two-month-old male C57BL/6N mice (purchased from Charles River Laboratories) were infused with CNP (Bachem, 0.05 μg/kg/min; ref. 20) or vehicle by subcutaneous osmotic minipumps. Systolic blood pressure and heart rate of the conscious mice were monitored by the tail-cuff method (17, 26). After 2 weeks, LV function was measured by invasive catheterization (see below and ref. 17). Plasma CNP was extracted with C18 columns and measured using a commercially available CNP-22 EIA Kit (Phoenix Pharmaceuticals) according to the manufacturer’s protocol.

### Genetic mouse model.

To achieve a cardiomyocyte-restricted deletion (KO) of GC-B, GC-B*^fl/fl^* mice (generated in our laboratory; ref. 26) were mated to transgenic mice expressing Cre-recombinase under the control of the αMHC-promoter *(αMHC-Cre^Tg^* mice) (25). Throughout all studies, CM GC-B–KO mice and corresponding GC-B*^fl/fl^* littermates (as controls) on a mixed C57BL6N/129Sv background were compared (all males, 2–4 months old). Genotypings were performed by genomic PCR using primers GC-B forward (5′-GGACGACCCATCCTGTGATA) and reverse (5′-GTTACAAACAAAAGCAAGATAAATACC), which amplify a 519 bp fragment for the GC-B (*Npr2*) WT allele, a 660 bp fragment for the floxed allele, and a 149 bp fragment for the knockout allele (26). Presence of the αMHC-Cre transgene was detected by PCR using primers Cre1 (5′-GCTGCCACGACCAAGTGACAGCAA) and Cre2 (5′-GTAGTTATTCGGATCATCAGCACAC) (400 bp band) (25). The hereby generated CM GC-B–KO mice (*GC-B^fl/fl^* *αMHC-Cre^Tg^*) and their control littermates (*GC-B^fl/fl^*) were kept under a 12-hour light/12-hour dark cycle at constant temperature (23°C) with unlimited access to food and water.

### Transverse aortic constriction.

Surgical TAC, for 3 or 14 days, or sham operations were performed as previously described (17, 25). In brief, 8-week-old mice with a body weight of approximately 25 g were anesthetized with isoflurane (2.5%), intubated, and put on a mechanical small-animal ventilator. The depth of anesthesia was checked by ensuring that noxious pinch stimulation of the hind paw, the forepaw, and the ear did not evoke any motor reflexes. After thoracotomy the aorta was ligated (7-0 prolene) between the innominate and left carotid artery with an overlying 27-gauge needle to generate a reproducible, discrete stenosis. After ligation, the needle was withdrawn. Sham mice underwent the same procedure without aortic ligation. Buprenorphine (0.05–0.1 mg/kg BW) was used for preemptive and postoperative analgesia (17).

### Hemodynamic studies.

Terminal closed-chest hemodynamic studies of aortic pressure and LV function were performed with a retrogradely inserted SPR-839 Mikro-Tip pressure-volume catheter (Millar Instruments) (17, 26). After 30 minutes of stabilization, calibrated pressure-volume loops were continuously recorded during 30 minutes under 2% isoflurane anesthesia; body temperature was kept at 37°C (17, 26). To determine the EDPVR, a transient inferior vena cava occlusion was then performed during simultaneous measurement of pressure/volume loops. EDPVR values were determined by fitting the end-diastolic points during vena cava occlusion using a linear fit: end-diastolic pressure = EDPVR × end-diastolic volume + intercept. Based on these linear fits, the average slope was calculated (EDPVR in mmHg/μL). Mice were sacrificed under deep anesthesia, the hearts were weighed, and left ventricles were dissected. LV slices were frozen in liquid nitrogen (for protein or mRNA extraction) or fixed in 4% buffered formaldehyde (for histology and immunohistochemistry).

### Histology, morphometry, and immunohistochemistry of cardiac sections.

LV paraffin-embedded sections were stained with periodic acid–Schiff (to discriminate cardiomyocyte cell borders), or 0.1% picrosirius red (for collagen) (17). Photomicrographs of the sections were evaluated using a computer-assisted image analysis system (Olympus), with the investigator blinded to the genotypes. The mean cross-sectional myocyte diameters were calculated by measuring approximately 100 cells with a centrally located nucleus per heart. Interstitial collagen fractions were obtained by calculating the ratio, as a percentage, between the collagen area and the total ventricular area in the corresponding section (17, 25).

For immunohistochemical studies a rabbit polyclonal antibody against CNP (HPA035362, 1:1500; MilliporeSigma) was used on 5 μm–thick paraffin sections. Antibody binding was detected by Rabbit Link (K1501, ready to use; Agilent Technologies) followed by CSAII Biotin-free Tyramide Amplification System (K1497; Agilent Technologies) and developed with DAB (Zytomed Systems GmbH). Negative controls were performed by using the appropriate immunoglobulin or by omitting the primary antibody.

### Experiments with cultured cardiomyocytes and fibroblasts.

Mice were sacrificed under deep isoflurane anesthesia and the hearts were rapidly cannulated. Cardiomyocytes and fibroblasts were isolated by liberase/trypsin digestion (protocol PP00000125 from The Alliance for Cellular Signaling) (17). Myocytes were plated for 4 hours on laminin-coated dishes. Fibroblasts were cultured in fibroblast growth medium (Promocell) during 5 days and then passaged once. For cGMP determinations the cells were incubated at 37°C with the phosphodiesterase inhibitor 3-isobutyl-1-methylxanthine (0.5 mM; MilliporeSigma) for 15 minutes and thereafter with synthetic CNP, ANP, or vehicle (saline) during an additional 10 minutes (17, 26). For studies of phospholamban and titin phosphorylation, myocytes were incubated with CNP for 10 minutes (17).

### Magnetic-assisted sorting of cardiac nonmyocytes.

Mice were sacrificed under deep isoflurane anesthesia and the hearts were rapidly cannulated. Hearts were retrogradely perfused with a liberase/trypsin solution as described above (17). The resulting single-cell suspension was centrifuged at 20*g* for 10 seconds to pellet rod-shaped cardiomyocytes (43) and passed through a 40 μm cell strainer. The supernatant containing the nonmyocyte fraction was incubated sequentially for 15 minutes with biotin-labeled anti-CD31 (catalog number 130-111-539, Miltenyi Biotec; for endothelial cells), anti–PDGFR-β (130-1089-866, Miltenyi Biotec; for pericytes), and anti–PDGFR-α antibodies (130.101.1905, Miltenyi Biotec; for fibroblasts) followed by 15 minutes’ incubation with anti-biotin magnetic microbeads (130-105-637, Miltenyi Biotec). Cells were separated from the cell suspension by magnetic-assisted sorting employing MS columns (130-042-201, Miltenyi Biotec) according to the manufacturer’s protocol (43).

### Gene expression studies.

Total RNA was isolated from isolated cardiac myocytes, fibroblasts, endothelial cells, and pericytes or LV myocardium using TRIzol reagent and 10 μg/mL Glycogen (Life Technologies, Thermo Fisher Scientific). After reverse transcription (Transcriptor First Strand cDNA Synthesis Kit; Roche), real-time RT-PCR was performed using a LightCycler Instrument (Roche) (26). For CNP expression studies, a preamplification step was performed with the TaqMan PreAmp Master Mix (Life Technologies, Thermo Fisher Scientific) using the CNP exon 2 and 3 spanning primers, GGCTTGTCCAAAGGCTGCT (forward) and TGTAAAAGCCACATTGCGTTG (reverse), according to the manufacturer’s instructions. The following primers and probes from Roche were used for RT-PCR: for CNP, AGCGGTCTGGGATGTTAGTG (forward), CGTTGGAGGTGTTTCCAGAT (reverse), and probe 75 (catalog 04688988001); for GC-B, TGTTTGGTGTTTCCAGTTTCC (forward), AGTTCTTCCCAGCGAATGC (reverse), and probe 67 (catalog 04688660001); for GC-A, TGGAGACACAGTCAACACAGC (forward), CCGAAGACAAGTGGATCCTG (reverse), and probe 60 (catalog 04688589001); for CD31, CATCATGGTCAACATAACAGAGC (forward), GATGGTGAAGTTGGCTACAGG (reverse), and probe 15 (catalog 04685148001); for PDGFR-β, GGTCACCGTGGTCCCACAT (forward) and CATCGGATCTCATAGCGTGGCT (reverse); and β_2_-Microglobulin served as reference gene, TACGCCTGCAGAGTTAAGCA (forward), GGTTCAAATGAATCTTCAGAGCA (reverse), and probe 117 (catalog 04693515001)).

### SDS-PAGE and immunoblot analyses.

Cardiomyocytes and whole left ventricles were solubilized in SDS or RIPA (for pro-CNP) sample buffer. The lysates were separated by 8%, 12%, or 15% PAGE followed by Western blot. The following targets were analyzed: pro-CNP (Thermo Fisher Scientific, PAS-47454; 1:1000); total and Ser_16_-phosphorylated phospholamban (Abcam, ab2865 and ab15000; both 1:1000); total and Ser_23/24_-phosphorylated troponin I (Cell Signaling Technology; 4002 and 4004; both 1:1000); and total as well as phosphorylated ERK1/2 (Cell Signaling Technology, 9102 and 9101; 1:1000). GAPDH (Cell Signaling Technology; 1:5000) was used to correct for loading (17).

To detect titin isoform composition and phosphorylation, cardiomyocytes and LV samples were solubilized in a modified Laemmli buffer (50 mM Tris-HCl at pH 6.8, 8 M urea, 2 M thiourea, 3% SDS *w/v*, 0.03% ServaBlue *w/v*, 10% *v/v* glycerol, 75 mM DTT). Samples were heated for 3 minutes at 96°C, centrifuged for 1 minute at 20°C at 4000*g*, and then separated by agarose-strengthened 1.8% SDS-PAGE (4, 5). Proteins were transferred to PVDF membranes. Blots were incubated with 3% BSA in Tween Tris-buffered saline for 1 hour at room temperature and thereafter with the primary antibodies overnight at 4°C. An anti–phospho-serine/threonine antibody (ECM Biosciences LLC; PP2551; 1:500) was used to assess total titin phosphorylation. The phospho-site–specific (phospho-Ser_4080_ and phospho-Ser_3991_) anti-titin antibodies were custom-made by Eurogentec with positions in the N2B unique sequence of mouse titin selected according to UniProtKB identifier [anti–phospho-N2Bus (Ser_3991_) antibody generated against EEGKS(PO_3_H_2_)LSFPLA (dilution 1:500); anti–phospho-N2Bus (Ser_4080_) antibody generated against LFS(PO_3_H_2_)EWLRNI (dilution 1:500)] (4, 5). The immunoreactive proteins were detected with secondary horseradish peroxidase–labeled goat anti-rabbit antibody (DakoCytomation; catalog number P0448 1:10,000) and enhanced chemiluminescence (ECL Western blotting detection; Amersham Biosciences). Immunoreactive signals were normalized to signals obtained from Coomassie-stained PVDF membranes referring to the entire protein amount transferred. The results were quantitated by densitometry (ImageQuant). Alternatively, all-titin phosphorylation was measured by PKGI-mediated back-phosphorylation as described (28). Briefly, skinned cardiomyocytes were phosphorylated with 1.68 × 10^−5^ U/μL purified PKGI (1 μg/mL; MilliporeSigma) and 300 μmol/L 8-pCPT-cGMP (a cGMP analog) (Calbiochem) for 60 minutes at 37°C in relaxing solution supplemented with phosphatase inhibitor cocktail (MilliporeSigma). The fibers were denatured and dissolved and titin immunoreactive bands visualized after 1.8% SDS-PAGE by incubating with the aforementioned antibodies directed against total Ser/Thr or Ser_4080_-titin (28).

### Single skinned CM preparations and passive force recordings.

Single demembranated LV cardiomyocytes (*n* = 16–24/4–5 cardiomyocytes/hearts) were prepared from unfrozen hearts and skinned in relaxing solution (for composition, see ref. 8) supplemented with 1% Triton-X-100. The tissue was washed in relaxing solution, and fiber bundles were dissected (diameter, 200 to 300 μm; length, 1.0 to 2.5 mm). Single cardiomyocytes were selected under an inverted microscope (Zeiss Axiovert 135, 40× objective; Carl Zeiss AG Corp), attached with silicone adhesive between a force transducer and a high-speed length controller (piezoelectric motor) as part of a Permeabilized Myocyte Test System (1600A; with force transducer 403A; Aurora Scientific) and studied at 20°C. Force and sarcomere length were recorded on stepwise stretching the samples from slack sarcomere length (average, 1.8 μm) to a maximum length of 2.4 μm (28). Another set of experiments was done after 30-minute preincubation with 1.68 × 10^−5^ U/μL purified PKGI (MilliporeSigma) and 300 μmol/L 8-pCPT-cGMP (Calbiochem).

### Statistics.

Results are presented as means ± SEM; sample sizes are provided in the legends to the figures. Analyses were performed using GraphPad Prism. Data were tested for normality (Shapiro-Wilk test) and equal variance (*F* test). For statistical analysis of 2 groups of parametric data, either 1- or 2-tailed Student’s *t* test was used; for nonparametric data, the Mann-Whitney *U* test was used. For multiple-group comparisons of normally distributed data, the 2-way ANOVA followed by the multiple-comparison Bonferroni’s *t* test was used. For data that were not normally distributed, the nonparametric Mann-Whitney *U* test was used for 2-group comparison, and the Kruskal-Wallis analysis was performed for multiple groups. For the qRT-PCR studies of endothelial versus pericyte markers in enriched cardiac cell fractions (normally distributed data), the 1-way ANOVA and Bonferroni’s *t* test were applied. For studies of cardiomyocyte passive stiffness, groups defined by levels of sarcomere length were compared in terms of continuous variables using either 1- or 2-tailed Student’s 2-sample *t* test (if distributional assumptions were satisfied) or Wilcoxon’s rank sum test (otherwise). All such tests were unpaired (the effect of all factors including stimulation length was assessed on independent samples) and stratified for equivalence on other factors. In all evaluations the significance level was set at *P* < 0.05.

### Study approval.

All animal studies were approved by the Animal Care and Use Committee of University of Würzburg (Regierung von Unterfranken, approval number 55.2 2532-2-135) and conformed with the *Guide for the Care and Use of Laboratory Animals* published by the US NIH (National Academies Press, NIH Publication No. 85-23, revised 2011) and the guidelines from Directive 2010/63/EU of the European Parliament on the protection of animals used for scientific purposes.

## Author contributions

KM, MH, FW, MA, KSS, MA, KS, SD, AM, HAB, and BVS conducted experiments and/or acquired, analyzed, and revised the data. NH and MK designed the research studies, analyzed and interpreted the data, and prepared the manuscript. All authors have approved the final version of the manuscript and agree to be accountable for all aspects of the work.

## Supplementary Material

supplemental data

supplemental Tables 1-3

## Version 1 10/15/2020

In-Press Preview

## Version 2 11/19/2020

Electronic publication
