# Supplementary material for: C-type natriuretic peptide moderates titin-based cardiomyocyte stiffness
Source: JCI Insight. 2020 Nov 19;5(22):e139910. doi: 10.1172/jci.insight.139910 (PMC7710274; doi:10.1172/jci.insight.139910)
Supplement: supplemental data [file jciinsight-5-139910-s020.pdf]

Supplemental Figure 1

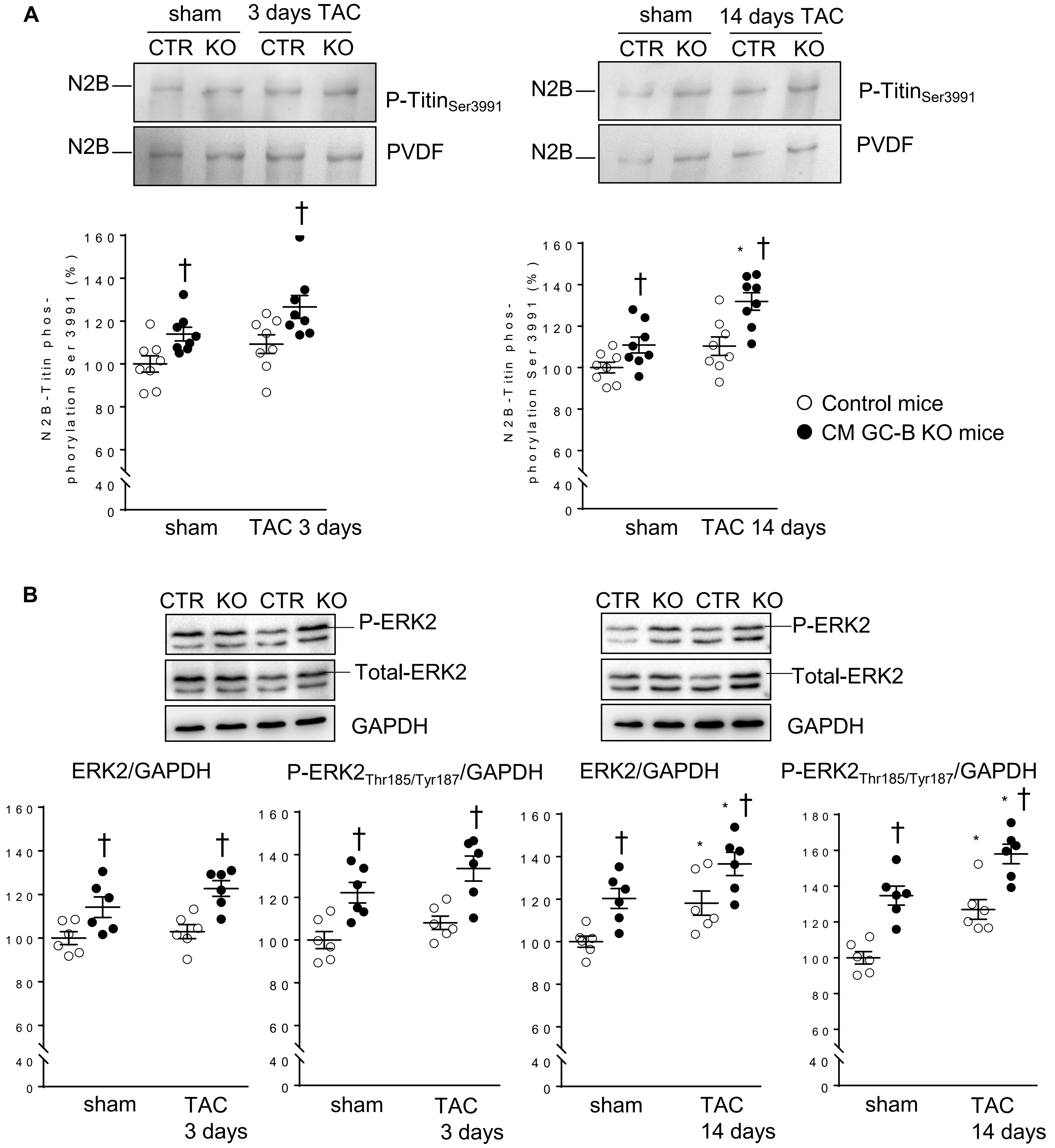

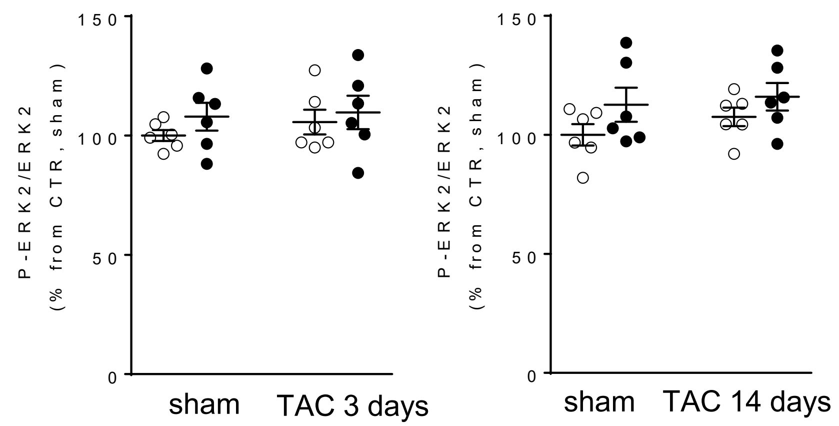

Supplemental Figure 1. Enhanced left ventricular levels of Ser_{3991}-phosphorylated titin as well as of total and phosphorylated ERK2 in CM GC-B KO mice. (A) Western blot analyses using a phospho-site specific antibody recognizing titin phosphorylated at Ser_{3991}. Levels were normalized to total titin (PVDF) and calculated as % from sham-operated control mice. **(B)** Western blot analyses using antibodies against total ERK2 and phosphorylated ERK2. Levels were normalized to GAPDH and calculated as % from sham-operated control mice. Bottom panels in (**B**): ratios of phosphorylated versus total ERK. Data are shown as mean ± SEM. *n* = 6-8 mice tested in duplicates. $^{*}P < 0.05$ versus sham; $^{\dagger}P < 0.05$ versus controls (*GC-B$^{fl/fl}$*) mice (2-way ANOVA).

Supplemental Figure 2

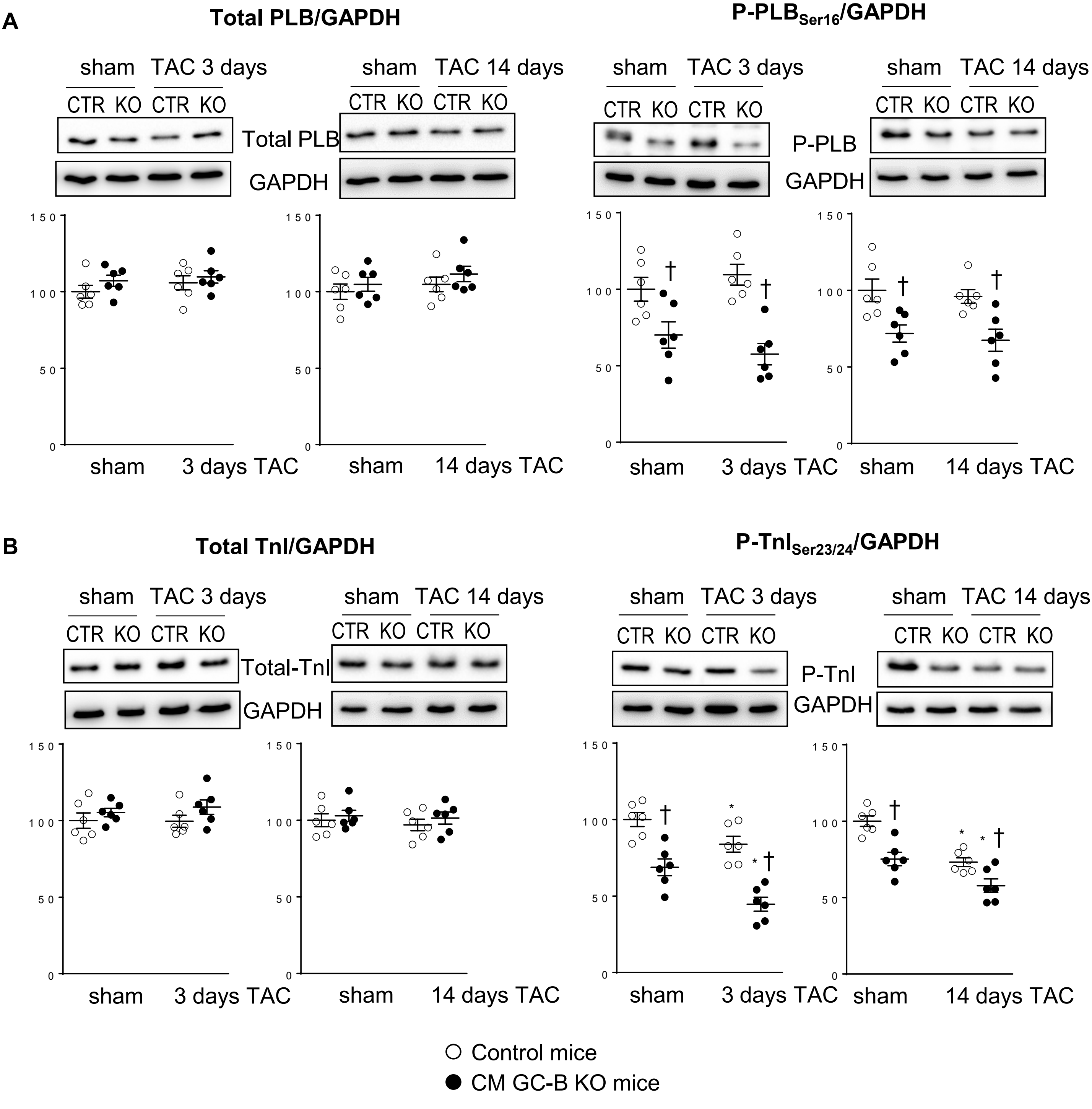

Supplemental Figure 2. Reduced left ventricular levels of Serine$_{16}$-phosphorylated phospholamban and Serine$_{23/24}$-phosphorylated troponin I in CM GC-B KO mice under sham and TAC conditions. (A) Western blot analyses using antibodies against total phospholamban (PLB, left panels) and phosphorylated PLB (P-Serine$_{16}$, right panels). **(B)** Western blot analyses using antibodies against total troponin I (TnI, left panels) and phosphorylated TnI (Serine$_{23/24}$, right panels). Target protein levels were normalized to GAPDH and calculated as percent (%) from sham-operated control mice. Means ± SEM. *n* = 6 mice per group tested in duplicates. **P* < 0.05 versus respective sham mice, †P < 0.05 versus control (*GC-B$^{fl/fl}$*) mice (2-way ANOVA).
