# Supplementary material for: C-type natriuretic peptide moderates titin-based cardiomyocyte stiffness
Source: JCI Insight. 2020 Nov 19;5(22):e139910. doi: 10.1172/jci.insight.139910 (PMC7710274; doi:10.1172/jci.insight.139910)
Supplement: supplemental Tables 1-3 [file jciinsight-5-139910-s021.pdf]

Supplemental Table 1. CNP infusion does not alter arterial blood pressure and left ventricular systolic and diastolic function under resting conditions.

	Vehicle	CNP
Plasma CNP, ng/ml	0.31 ± 0.05	0.54 ± 0.09*
**Blood pressure (tail cuff, awake mice)**		
SBP, mmHg	111 ± 3	109 ± 3.2
DBP, mmHg	75 ± 3	73 ± 6.1
**LV function (invasive catheterization, anesthetized mice)**		
Heart rate, bpm	506 ± 17	490.7 ± 24
Ejection fraction, %	49 ± 2.3	51 ± 3.6
dP/dt_{max}, mmHg/sec	10348 ± 880	9784 ± 1077
dP/dt_{min} , mmHg/sec	9570 ± 654	9030 ± 963
Stroke volume, µl	14.1 ± 0.8	14.3 ± 0.34
End-diastolic Vol, µl	28.1 ± 1.9	28.2 ± 2.1
End-systolic Vol, µl	16.8 ± 1.2	15.0 ± 1.9
EDPVR, mmHg/µl	0.48 ± 0.6	0.43 ± 0.1

Cardiovascular parameters of vehicle and CNP-treated C57Bl6 mice. Systolic (SBP) and diastolic (DBP) blood pressure of trained mice was measured by tail-cuff plethysmography (6 mice per group). Left ventricular function of anesthetized mice was measured by invasive haemodynamics (8 mice per group). Tested with Student t test.

Supplemental Table 2. Mice with cardiomyocyte-restricted deletion of the GC-B receptor (CM GC-B KO) have unaltered blood pressure and left ventricular systolic and diastolic function under resting conditions.

	Control mice	CM GC-B KO mice
**Blood pressure (tail cuff, awake mice)**		
SBP, mmHg	106 ± 4	107 ± 4
DBP, mmHg	74 ± 6	77 ± 6
**LV function (invasive catheterization, anesthetized mice)**		
Heart rate, bpm	557 ± 10	566 ± 7
Ejection fraction, %	58 ± 3.3	64 ± 1.6
dP/dt_{max}, mmHg/sec	10530 ± 180	11525 ± 430
dP/dt_{min}, mmHg/sec	10896 ± 427	11835 ± 363
Stroke volume, µl	14.6 ± 1.3	13.3 ± 2.8
End-diastolic Vol, µl	24.0 ± 1.3	21.0 ± 4.1
End-systolic Vol, µl	12.8 ± 0.2	11.0 ± 1.2
EDPVR, mmHg/µl	0.44 ± 0.4	0.38 ± 0.09
**Necropsy**		
Body weight, g	23.7 ± 0.5	23.9 ± 0.9
Heart weight, mg	121.2 ± 1.7	121.3 ± 4.5
Tibia length, mm	1.8 ± 0.02	1.78 ± 0.02

Baseline cardiovascular parameters of control and CM GC-B KO mice. Systolic (SBP) and diastolic (DBP) blood pressure of trained awake control and CM GC-B KO mice was measured by tail-cuff plethysmography (7 mice per group). Left ventricular function of anesthetized mice was measured by invasive haemodynamics (4 mice per group). Tested with Student t test

	Sham		TAC 3 days		TAC 2 weeks	
	**CTRL (n = 14)**	**CM GC-B KO (n = 13)**	**CTRL (n = 11)**	**CM GC-B KO (n = 14)**	**CTRL (n = 17)**	**CM GC-B KO (n = 19)**
**Aorta**						
Max Aortic Pressure (mmHg)	112.6 ± 1.4	111.0 ± 2.3	147.2 ± 4.2*	142.8 ± 4.4*	158.2 ± 4.0*	160.0 ± 4.8*
**Left Ventricle**						
Cardiac Output (µl/min)	8618.5 ± 537.6	9365.2 ± 341.2	7090 ± 865.1	6749.6 ± 414.7*	6816.8 ± 551.9	7099.6 ± 448.6*
Stroke Volume (µl)	16.50 ± 0.95	17.47 ± 0.69	12.32 ± 1.65	11.42 ± 0.70*	12.43 ± 1.02	12.22 ± 1.09*
P_{max} (mmHg)	109.7 ± 1.7	108.5 ± 2.5	141.7 ± 4.3*	136.1 ± 4.7*	159.0 ± 4.4*	155.5 ± 3.5*
Heart rate (bpm)	527.8 ± 12.3	543.1 ± 14.5	584.7 ± 15.6	593.1 ± 9.8	555.6 ± 14.3	579.5 ± 10.1
Ejection fraction (%)	56.19 ± 3.18	55.96 ± 2.50	38.16 ± 5.53	27.41 ± 2.42*	49.65 ± 3.18	37.23 ± 2.56*
Tau (msec)	6.06 ± 0.25	5.85 ± 0.21	8.45 ± 0.90*	9.14 ± 0.54*	7.29 ± 0.43	8.15 ± 0.44*

Supplemental Table 3: **Hemodynamic parameters of control and CM GC-B KO mice after 3 or 14 days of TAC or sham operation**. Data are shown as mean ± SEM. *n* = 11–19/group. *P < 0.05 vs respective sham mice. Aortic pressure (proximal to the surgical stenosis), LV P_{max} and Tau were evaluated by 2-way ANOVA with Bonferroni multiple comparison t test; the other parameters were tested with Kruskal Wallis Analysis.
